# Prolonged P wave duration predicts stroke mortality among type 2 diabetic patients with prevalent non-major macrovascular disease

**DOI:** 10.1186/1471-2261-14-168

**Published:** 2014-11-25

**Authors:** Teemu Vepsäläinen, Markku Laakso, Seppo Lehto, Auni Juutilainen, Juhani Airaksinen, Tapani Rönnemaa

**Affiliations:** Department of Medicine, University of Turku and Turku University Hospital, Kiinamyllynkatu 10, 20520 Turku, Finland; Department of Medicine, University of Eastern Finland and Kuopio University Hospital, Puijonlaaksontie 2, 70211 Kuopio, Finland

**Keywords:** Type 2 diabetes, Cardiovascular disease, ECG, P wave duration, Stroke mortality

## Abstract

**Background:**

Prolonged P wave duration is a marker of delayed inter-atrial conduction which may predict cardiovascular disease (CVD). Type 2 diabetes is a risk factor for all atherosclerotic manifestations including stroke. We evaluated the prognostic significance of prolonged P wave duration among middle-aged Finnish type 2 diabetes patients with and without prevalent non-major macrovascular disease (PNMMVD) with respect to total and stroke mortality.

**Methods:**

We followed up for 18 years 739 type 2 diabetic patients without previous major CVD event at baseline. Participants were stratified according to P wave duration (<114 or ≥114 ms) and PNMMVD (i.e. coronary heart disease defined as ischaemic ECG changes and typical symptoms of angina pectoris, or claudication; yes or no). The Cox proportional hazards model was used to estimate the joint association between P wave duration, PNMMVD and the mortality risk.

**Results:**

During the follow-up, 509 patients died, and 59 of them died from stroke. Those who had prolonged P wave duration had 2.45 (95% confidence interval: 1.11-5.37) increased stroke mortality among PNMMVD patients. In patients without PNMMVD, there was no relationship between P wave duration and stroke mortality.

**Conclusions:**

As an easily measurable factor P wave duration merits further studies with higher number of patients to evaluate its importance in the estimation of stroke risk in type 2 diabetic patients with PNMMVD.

## Background

P wave duration is a marker of atrial conduction derived from standard electrocardiogram (ECG). Prolonged P wave duration has been suggested to be an easily measurable risk factor for underlying subclinical heart disease [1]. In fact, it has been associated with atrial fibrillation (AF) and also more recently with increased all-cause, cardiovascular disease (CVD) and stroke mortality among general population [1–4]. Prolonged P wave duration is also associated with diabetes [1].

In patients with type 2 diabetes, atherosclerosis is more common and the mortality is higher after cardiovascular events such as acute myocardial infarction (MI), stroke, or lower extremity amputation due to peripheral vascular disease [5] than in their non-diabetic counterparts. To our knowledge there are no previous long-term studies studying the association between P wave duration and stroke mortality among type 2 diabetes patients, especially among diabetes patients with high risk for CVD events. Therefore, we decided to evaluate the prognostic significance of prolonged P wave duration with respect to stroke mortality among middle-aged type 2 diabetes patients with or without signs of non-major macrovascular disease (coronary heart disease without myocardial infarction, or claudication). We contemplated that patients having signs of non-major macrovascular disease are in high risk of developing major CVD events and aimed to examine whether among these patients prolonged P wave duration would identify those who are in particularly high risk for stroke mortality.

## Methods

A detailed description of the study participants has been published previously [6]. Briefly, 1059 subjects (581 men, 478 women) with type 2 diabetes, aged 45–64 years, born and living in the Turku University Hospital district in West Finland or in the Kuopio University Hospital district in East Finland were identified through a national drug reimbursement register. Patients with type 1 diabetes, based on early onset diabetes, history of ketoacidosis and low glucagon-stimulated C-peptide measurement at baseline, were excluded.

We excluded from all statistical analyses a total of 320 subjects. Exclusion criteria were major previous CVD event (possible or definite stroke, possible or definite MI or lower-extremity amputation) (n = 252) or missing ECG data (n = 108) or known AF (n = 23). Thus, the final study population comprised 739 patients with type 2 diabetes (381 men, 358 women).

### Baseline study

The baseline examination was carried out between 1982 and 1984 during one outpatient visit at the Clinical Research Unit of the University of Kuopio or the Rehabilitation Research Centre of the Social Insurance Institution in Turku. The visit included an interview to determine history of smoking, alcohol intake, physical activity, use of medication and history of chest pain suggestive of coronary heart disease (CHD). The examinations and the methods used have been described previously in detail [6].

On all participants at baseline a standard 12-lead resting ECG was performed at 8–9 o’clock AM. Before recording the subject had fasted for 12 hours. ECG abnormalities were recorded according to the Minnesota code, including left ventricular hypertrophy (LVH). The coder of the ECGs was blinded to the glucose tolerance status and other information on the study subjects. The ischemic changes by ECG (The Whitehall criteria) included Minnesota codes 1.1-1.3, 4.1-4.3, 5.1-5.3, 7.1 [7]. In addition, a three-dimensional computerized ECG recording was performed. Measurements for atrial conduction were based on the Dalhousie ECG Program v. 8.0 [8]. The program was implemented on a SM-4 computer and adapted for use by the ECG terminals and data communication equipment (Kone 620, Kone Oy, Finland). P wave duration was measured from the onset of the P wave to the end of P wave from the spatial magnitude curve of the X, Y, Z components after selective averaging. Computer-identified time points including P wave onset point were displayed, visually verified and obvious measurement errors corrected by one of the authors (M.L.). The Rose classification was used to evaluate the presence of typical angina pectoris and intermittent claudication [7]. Prevalent non-major macrovascular disease (PNMMVD) was defined as coronary heart disease without definite or possible myocardial infarction (ischaemic ECG changes and typical symptoms of angina pectoris), or claudication (yes or no).

Medical records of patients who reported that they had been admitted to hospital for chest pain were reviewed by two investigators (M.L. and T.R.) after careful standardization of the methodology. World Health Organization (WHO) criteria for verified definite or possible MI based on chest pain symptoms, ECG changes and determination of enzyme activities were used to define previous MI [9]. WHO criteria were also used to define previous definite or possible stroke [10]. Non-traumatic lower extremity amputations were recorded. Blood pressure was measured with the person in the sitting position after a 5-min rest. Body mass index (BMI) was calculated by dividing weight in kilograms divided by the square of height in meters. Hypertension was defined as systolic blood pressure ≥160 mmHg, or diastolic pressure ≥95 mmHg, or antihypertensive drug treatment.

### Biochemical methods

After 12-h fast, blood was collected at 08.00. Glycosylated haemoglobin (HbA_1_) level (reference range in non-diabetic subjects 5.5–8.5%) was determined by affinity chromatography (Isolab, Akron, OH, USA). Levels of serum total cholesterol, HDL cholesterol and triglycerides were determined using standard laboratory methods [6]. Coomassie brilliant blue was used to measure total urinary protein concentration from a morning spot urine specimen [11]. The interassay coefficient of variation was in this study was 7% at protein levels of 100 and 250 mg/L and 3% at 600 mg/L.

### Follow-up study

Follow-up continued for 18 years until 1 January 2001. Information on vital status of all participants and copies of death certificates of subjects who had died before the end of follow-up were obtained from the national Cause-of-Death Register (Statistics Finland). All death certificates of participants were reviewed by two of the authors (A.J. and S.L.). In the final classification of causes of death, hospital records and autopsy records were also reviewed if available. The study endpoints were total mortality, CVD mortality [International Classification of Diseases 9th revision (ICD-9) codes 390–459], CHD mortality (ICD-9 codes 410–414) and stroke mortality (ICD-9 codes 431–438).

The Ethics Committees of the Turku University and Turku University Central Hospital, and the University of Kuopio approved the study. Informed written consent was obtained from all participants.

### Statistical analyses

All statistical analyses were performed using PASW statistics (version 18.0; SPSS Inc., Chicago, IL, USA). Data for continuous variables are expressed as mean ± SD or median (interquartile range) and categorical variables as percentage. Baseline characteristics were compared using analysis of variance for continuous variables and the chi-square test for categorical variables. Because of a skewed distribution, total triglycerides and urinary protein was analysed after logarithmic transformation.

There is still controversy for the cut off level for prolonged P wave duration [12, 13]. Various cut off values have been suggested for prolonged P wave duration, such as 110 ms or 120 ms, where most of the recommendations are based on earlier textbooks and other publications that often did not involve original work [12, 13]. Therefore, we have made analyses using several cut off values for P wave duration, 104 ms, 108 ms, 110 ms, 114 ms and 120 ms (corresponding to 50, 60, 70, 80 and 90 percentiles of the distribution) to find out a threshold of P wave duration associated with a steep increase in mortality. Death rates/1000 patient years according to 50, 60, 70, 80 and 90 percentiles of P wave duration were as follows; for total mortality: 58.9, 59.6, 60.6, 64.7, 75.4, and for stroke mortality: 6.7, 7.7, 8.1, 9.1 and 11.6. For lower percentiles mortality risk increases relatively smoothly but after 80th percentile the risk increased more rapidly. Therefore we decided to use the 80 percentile cut off value to categorize participants according to P wave duration into two groups; normal or prolonged (<114 ms or ≥114 ms). This cut off value for P wave duration was used to calculate hazard ratios for mortality among patients with and without PNMMVD.

We used the Cox proportional hazards model to evaluate the association of prolonged P wave duration among patients with or without PNMMVD on total, CVD and stroke mortality with P wave duration <114 ms as the reference. Unadjusted and adjusted hazard ratios and their 95% confidence intervals were calculated. Adjustment was made for age, sex, duration of diabetes, area of residence (East or West Finland), total cholesterol, use of alcohol (user vs. non-user), smoking (smoker vs. non-smoker), HDL cholesterol, total triglycerides (log), urinary protein (log), HbA_1_, diabetes medication (diet alone, oral drugs, insulin), physical activity, hypertension, BMI, heart rate and LVH. Kaplan–Meier procedure was used to evaluate the associations between P wave duration and stroke mortality in participants with and without PNMMVD. There were no interactions in Cox proportional hazards model between sex and P wave duration or sex and PNMMVD with respect to mortality. Therefore, men and women were combined in all statistical analyses. *P* < 0.05 was considered to be statistically significant.

## Results

PNMMVD stratification was made because we observed that total and stroke mortality was significantly increased among patients with PNMMVD: 76.8% (322 out of 419) of patients with PNMMVD and 58.4% (187 out of 320) of patients without PNMMVD died during follow-up (P <0.001 log rank). The corresponding figures for stroke mortality were 9.1% (38 out of 419) and 6.6% (21 out of 320), respectively (p = 0.02 log rank).

We observed interaction between P wave duration 114 ms (= 80th percentile) and PNMMVD with respect to stroke mortality.

The clinical and laboratory characteristics of the participants at baseline according to P wave duration are presented in Table 1. Prolonged P wave duration at baseline was associated with male gender, increased age, triglycerides, BMI, increased urinary protein. Prolonged P wave duration was also associated with higher prevalence of hypertension and PNMMVD. A total of 16.1% of the subjects had prolonged P wave duration ≥114 ms and PNMMVD, 6.9% had P wave ≥114 ms, but no PNMMVD. 40.6% had P wave <114 ms and PNMMVD, and furthermore 36.4% had P wave <114 ms and no PNMMVD. PNMMVD patients’ median P wave duration was 106 ms (25th percentile: 96 ms, 75th percentile: 110 ms), minimum 62 ms and maximum 174 ms. Patients without PNMMVD had median P wave duration of 104 ms (25th percentile: 98 ms, 75th percentile: 114 ms), minimum 70 ms, maximum 135 ms.

**Table 1:** **Baseline clinical and laboratory characteristics and the number of subjects with various outcomes according to P wave duration among type 2 diabetes patients**

	P wave duration		
Variables	<114 ms	≥114 ms	P
N	569	170	
Age (years)	57.5 ± 5.29	59.0 ± 4.21	0.001
Duration of diabetes (years)	7.9 ± 3.9	8.0 ± 3.9	0.649
Total cholesterol, mmol/L	6.68 ± 1.66	6.81 ± 1.98	0.381
HDL cholesterol, mmol/L	1.25 ± 0.363	1.19 ± 0.310	0.051
Triglycerides, mmol/L	2.38 ± 2.58	3.10 ± 4.08	0.006
Glycosylated hemoglobin A_1_, %	9.90 ± 2.39	10.1 ± 1.93	0.257
BMI, kg/m^2^	29.0 ± 5.23	30.2 ± 5.13	0.007
Urinary protein, mg/L	220 ± 445	396 ± 879	0.001
Physical activity, MET^†^	4.1 ± 1.9	3.85 ± 1.8	0.091
Heart rate, bpm	74 ± 13	75 ± 15	0.368
Women, %	50.6	41.2	0.031
Current smokers, %	16.7	17.6	0.772
Hypertension/-medication, %	56.6	74.7	<0.001
Systolic BP, mmHg	153 ± 23	157 ± 23	0.036
Diastolic BP, mmHg	85 ± 12	88 ± 12	0.005
Alcohol users, %	35.9	43.5	0.070
Area of residence, Turku, %	51.8	48.8	0.489
LVH, %	12.3	16.5	0.160
PNMMVD*, %	52.7	70.0	<0.001
Diabetes treatment			0.823
diet only, %	13.5	14.7	
oral drugs, %	74.2	71.8	
insulin therapy, %	12.3	13.5	
Death rates/1000-patient-years			
Total mortality	52.9	64.7	0.039
CVD mortality	33.7	43.5	0.070
CHD mortality	24.7	30.3	0.326
Stroke mortality	5.7	9.1	0.153

Data are expressed as the mean ± SD, unless otherwise indicated.

*Prevalent non-major macrovascular disease (ischaemic ECG changes and typical symptoms of angina pectoris, or claudication), ^†^Metabolic equivalent task.

### Outcome according to P wave duration and PNMMVD

**Figure 1:**
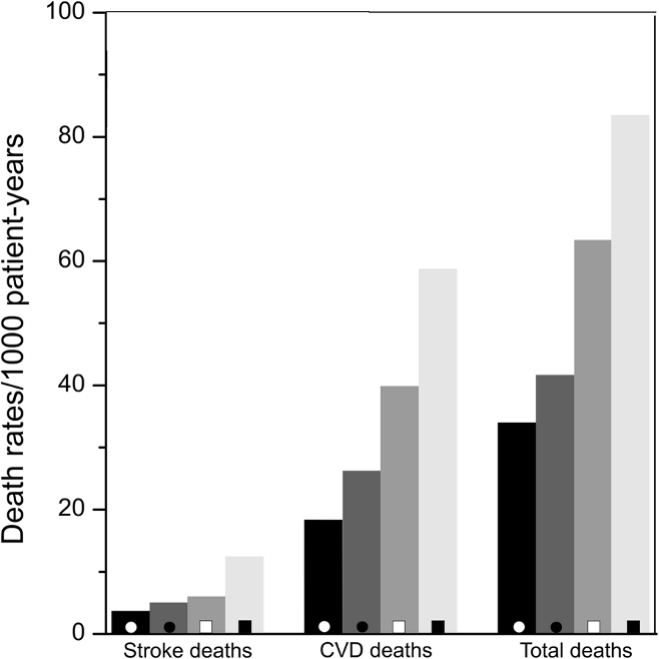
**Total, CVD and stroke mortality according to P wave duration stratified by prevalent non-major macrovascular disease (PNMMVD): ○ No PNMMVD with P wave duration ≥114 ms; ● No PNMMVD with P wave duration <114; □ PNMMVD with P wave duration <114 ms; ■ PNMMVD with P wave duration ≥114 ms.** Event-rates are expressed per 1000 patient- years of follow-up. Number of patients without PNMMVD in the two P wave duration groups (<114 ms and ≥114 ms) were 269 and 51, respectively; the respective number of patients with PNMMVD were 300 and 119.

During 9,185 patient-years of follow-up a total of 509 (68.9%) patients died, 329 (44.5%) died from CVD and 59 (8.0%) died from stroke. Among the participants without prolongation of the P wave duration, 221 out of 300 with PNMMVD died (144 from CVD and 22 from stroke) and 160 out of 269 participants without PNMMVD died (99 from CVD and 19 from stroke). Among the participants with prolongation of the P wave duration, 101 out of 119 with PNMMVD died (72 from CVD and 16 from stroke) and 27 out of 51 participants without PNMMVD died (14 from CVD and 2 from stroke). The event rates of total, CVD and stroke mortality per person years according P wave duration in patients with or without PNMMVD are shown in Figure 1.

Among PNMMVD patients those who had prolonged P wave duration had significantly an increased risk for total, CVD and stroke mortality in univariate analysis. This trend was also seen in multivariate analysis, but the association was significant only for stroke mortality. The relative risk for stroke mortality among PNMMVD patients with prolonged P wave duration was 2.25-fold (CI 1.18-4.31) increased in univariate and 2.45-fold (CI 1.11-5.37) increased in multivariate analysis when compared to patients with normal P wave duration (Table 2). Among patients without PNMMVD prolonged P wave duration was not associated with increase in total, CVD or stroke mortality. In fact prolonged P wave duration with respect to CVD mortality was associated with reduction of mortality.

**Table 2:** **Hazard ratios (P wave ≥114 ms vs. P wave <114 ms) for total mortality, CVD and stroke mortality stratified by prevalent non-major macrovascular disease (PNMMVD)**

	HR (95% CI)			
	Age-adjusted hazard ratios		Multivariate-adjusted hazard ratios	
Variables	P wave ≥114 vs. <114 ms	P for interaction	P wave ≥114 vs. <114 ms	P for interaction
Total mortality				
No PNMMVD	0.75 (0.50-1.13)		0.70 (0.45-1.09)	
		0.020		0.047
PNMMVD	1.31 (1.03-1.66)*		1.18 (0.90-1.54)	
CVD mortality				
No PNMMVD	0.63 (0.36-1.10)		0.22 (0.27-0.90)*	
		0.010		0.015
PNMMVD	1.45 (1.09-1.93)*		1.32 (0.95-1.84)	
Stroke mortality				
No PNMMVD	0.47 (0.11-2.04)		0.36 (0.08-1.66)	
		0.057		0.089
PNMMVD	2.25 (1.18-4.31)*		2.45 (1.11-5.37)*	

*P < 0.05 for the difference between P wave ≥114 vs. <114 ms. Variables in multivariate adjusted: age, sex, area of residence, diabetes duration, total cholesterol, HDL cholesterol, triglycerides(log), proteinuria(log), smoking, alcohol, HbA_1_, presence of hypertension, BMI, type of diabetes therapy, physical activity, heart rate and left ventricular hypertrophy.

**Figure 2:**
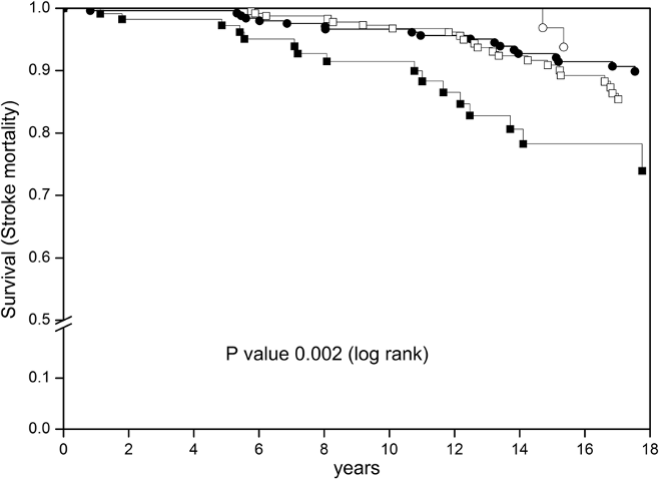
**The Kaplan-Meier survival curve for stroke mortality according to P wave duration stratified by prevalent non-major macrovascular disease (PNMMVD): ○ No PNMMVD with P wave duration ≥114 ms (n = 51); ● No PNMMVD with P wave duration <114 (n = 269); □ PNMMVD with P wave duration <114 ms (n = 300); ■ PNMMVD with P wave duration ≥114 ms (n = 119).** *P* value denotes the difference between the survival curves (log rank).

The Kaplan-Meier curve (Figure 2) indicates the cumulative survival with respect to stroke mortality for patients with normal or prolonged P wave duration stratified by baseline PNMMVD. Patients with PNMMVD and P wave duration ≥114 ms had poorer prognosis than those with P wave duration <114 ms. This started to become evident after four years of follow-up. Among patients without PNMMVD there was no statistical difference between P wave duration curves in Kaplan-Meier analysis.

## Discussion

As far as we know, there are no previous studies aiming to investigate the predictive value of prolonged P wave duration on stroke mortality in diabetic subjects with or without PNMMVD. In our prospective study of middle aged Finnish type 2 diabetes patients, prolonged P wave duration was significantly associated with increased stroke mortality rate among patients with PNMMVD. This association was independent of conventional CVD risk factors, proteinuria, duration of diabetes, diabetes treatment, glycemic control, heart rate and LVH. In patients without PNMMVD prolonged P wave duration was not associated with stroke mortality.

P wave duration is a marker of atrial conduction derived from ECG. Normal P wave can be considered to be less than 110 ms [12–14]. Prolonged P wave duration signifies conduction delay between right and left atrium due to impulse slowing or blockage, probably most often but not exclusively in the Bachmann bundle. On the ECG this conduction delay is referred to as interatrial block (IAB) [14–16]. IAB is one of the most common ECG abnormalities but remarkably underdiagnosed [17–20]. The exact underlying cause for IAB remains relatively unknown. However, atrial tissue sampling from patients with IAB consistently shows intercellular fibrotic changes and intracellular inclusions, particularly in the sarcomere and sarcoplasmic reticulum [21]. Coronary artery disease and traditional cardiovascular risk factors, such as hypertension, hypercholesterolemia, smoking, obesity, physical inactivity and higher age have been associated with IAB [22]. Similar associations were also seen in our study with respect to prolonged P wave duration. It is possible that these CVD risk factors increase P wave duration via endothelial injury, leading to ischemia-induced inter-atrial conduction delay [22].

Even though our study has limited statistical power because of the smaller number of patients in the prolonged P wave groups, there is two possibly inter-related mechanisms suggesting that our findings are likely to be valid and that these mechanisms could explain the excess stroke mortality among PNMMVD type 2 diabetes patients with prolonged P wave duration, i.e. IAB. First, IAB is associated with left atrial enlargement (LAE) and left atrium (LA) electromechanical dysfunction [23, 24]. In one study, patients with IAB had lower LA emptying fraction, lower LA stroke volume and lower LA kinetic energy [24]. The degree of these abnormalities was related to the severity of interatrial conduction delay represented by P wave duration [24]. These changes in LA could enhance the risk for thrombosis. In fact, recently it was noticed that patients who had embolic stroke had significantly higher prevalence of IAB on the ECG [2, 3]. Moreover, in recent study prolonged P wave duration was also associated with increased all-cause and CVD mortality [1]. Secondly, IAB has also been associated with AF [2]. The deterioration of interatrial conduction in IAB results in a shorter wavelength and this combined with the probable LAE could increase the number of wavelets in the atrium [25, 26]. These mechanisms could enhance the risk that AF would occur and sustain itself and furthermore increase the risk for stroke mortality among these patients. In our study, only patients with PNMMVD had increased stroke mortality when P wave duration was ≥114 ms. It is probable that those patients had more advanced coronary heart disease and therefore, had more severe LA electromechanical dysfunction and were more prone to AF.

It is also possible that independent of an increased risk for atrial thrombus formation, prolonged P wave duration is a marker of more advanced atherosclerosis and therefore represents patients who more likely have widespread vascular damage affecting arteries, also in the brain.

Our study has some limitations. It is probable that we could not exclude from the final analysis all patients who had previous MI at baseline because more precise imaging techniques were not available at baseline. Secondly, we were able to examine only total stroke mortality and not able to separate between thromboembolic and haemorrhagic stroke mortality. In the PNMMVD category we were not able to include specifically patients with carotid or cerebrovascular atherosclerosis, however it is plausible that patients included in this category had already more general arterial disease and therefore, were also likely to have carotid or cerebrovascular atherosclerosis. Thirdly, we had ECG recording and risk factor measurements only at baseline. Therefore, we have no data on changes in the P wave duration or other risk factors during the follow-up. Furthermore, by design of our study, we had only P wave duration as a marker of atrial conduction. More complete assessments of the P wave, e.g., P wave dispersion and amplitude, would provide deeper insight into the association between P wave characteristics and mortality among patients with type 2 diabetes and PNMMVD. Nevertheless, and to support our results, recently only P wave duration has been associated with CVD and stroke mortality [1–3]. Furthermore, we cannot conclude which ECG lead would be the most accurate lead to measure P wave duration. However, it has been suggested that P wave duration should be measured from all the leads and the lead giving the longest duration should be used [11]. Our study results are in accordance with this suggestion.

Finally, at the time of our baseline examination, risk assessments and interventions for CVD and stroke mortality in type 2 diabetes patients were not routinely performed, in contrast to the situation in clinical practice today. Diabetes and conventional CVD risk factor control has become better and procedures to treat acute cardiac and stroke events have evolved and become readily available since the baseline study. Therefore, the prognosis of patients with diabetes and diabetic complications is probably better than it was some 20 years ago. Therefore, it is not possible to draw final conclusions on how strong the association between P wave duration and stroke mortality is today. Due to these limitations, it is likely that our study rather underestimates than overestimates the association of P wave duration and PNMMVD with stroke mortality.

## Conclusions

Middle-aged type 2 diabetes patients with PNMMVD and prolonged P wave duration had increased risk for stroke mortality in our study. Among patients without PNMMVD, P wave duration was not associated with stroke mortality. Further studies with higher number of patients are needed to determine the clinical value of measuring P wave duration in the estimation of stroke risk in type 2 diabetic patients with prevalent non-major macrovascular disease.
